# Peripheral Rhegmatogenous Retinal Detachment in Straatsma Syndrome: A Case Report

**DOI:** 10.1155/crop/6341643

**Published:** 2026-04-16

**Authors:** Isabela Nunes de Sousa Bandeira Lima, Alline Gonçalves dos Reis Melo, Wener Passarinho Cella

**Affiliations:** ^1^ Department of Medicine, Dom Bosco University Center (UNDB), São Luís, Maranhão, Brazil; ^2^ Department of Ophthalmology, Ophthalmological Reference Hospital (Vision One), São Luís, Maranhão, Brazil

**Keywords:** case report, myelinated retinal nerve fibers, rhegmatogenous retinal detachment, Straatsma syndrome, vitreoretinal interface

## Abstract

**Introduction:**

Straatsma syndrome is a rare congenital condition classically defined by the triad of persistent myelinated retinal nerve fibers, ipsilateral axial myopia, and amblyopia. Although generally regarded as a stable developmental anomaly, growing evidence suggests that eyes with Straatsma syndrome may harbor clinically relevant abnormalities of the vitreoretinal interface, predisposing them to late‐onset retinal detachment. Most reported cases describe macular hole–associated retinal detachment driven by focal, abnormally strong vitreoretinal adhesion overlying myelinated fibers at the macula. Peripheral rhegmatogenous mechanisms, however, remain poorly characterized.

**Case Presentation:**

We report a 64‐year‐old man with longstanding unilateral Straatsma syndrome who presented with sudden, severe visual loss. Fundus examination and ultrasonography revealed a macula‐involving rhegmatogenous retinal detachment originating from a superonasal peripheral retinal tear, without evidence of macular hole formation. Intraoperatively, pars plana vitrectomy disclosed unusually firm vitreoretinal adhesion at the site of the peripheral break, despite the absence of visible myelinated nerve fibers in that region. The patient underwent combined phacoemulsification, pars plana vitrectomy, scleral buckling, and silicone oil tamponade, achieving complete retinal reattachment. Postoperatively, chronic cystoid macular edema developed and was successfully treated with silicone oil removal and intravitreal dexamethasone implantation. Final best corrected visual acuity improved to 20/60, consistent with the pre‐existing amblyopia.

**Conclusion:**

This case documents a peripheral, tear‐related rhegmatogenous retinal detachment in Straatsma syndrome, a mechanism distinct from the macular hole–driven pathway most commonly reported. Although the coexisting high axial myopia constitutes a well‐established independent risk factor for peripheral retinal breaks, the intraoperative observation of unusually firm vitreoretinal adhesion at a site devoid of visible myelinated fibers raises the hypothesis that Straatsma syndrome may involve a diffuse abnormality of the vitreoretinal interface extending beyond areas of clinically apparent myelination. As a single observational case, these findings are hypothesis‐generating and cannot establish causality; nevertheless, they underscore the importance of meticulous peripheral retinal examination and long‐term surveillance in affected patients, particularly in the presence of high axial myopia.

## 1. Introduction

Persistent retinal myelinated nerve fibers represent a developmental anomaly characterized by the ectopic presence of oligodendrocytes and myelin sheaths within the retinal nerve fiber layer. Autopsy‐based studies estimate its prevalence at approximately 1% of the general population [1]. Although most cases are isolated and clinically benign, extensive retinal myelination may constitute Straatsma syndrome, a rare clinical entity first described in 1979. This syndrome is classically defined by a unilateral triad consisting of persistent and extensive myelination of the retinal nerve fiber layer, axial myopia, and amblyopia [2]. The pathophysiology of Straatsma syndrome remains incompletely understood. However, it has been postulated that failure of the lamina cribrosa to function as an effective barrier allows anomalous migration of oligodendrocytes into the retina during prenatal development. This aberrant migration results in opaque myelinated plaques that obscure retinal vessels and impair visual quality [1, 3].

Clinically, Straatsma syndrome is regarded as a relatively stable condition, with little to no progression in the extent of retinal myelination over time. Nevertheless, the associated amblyopia is typically refractory to conventional occlusion therapy, largely due to the combined effects of marked anisometropia and structural visual deprivation. In addition, extensive myelinated nerve fibers may contribute to axial elongation of the globe, further exacerbating anisometropia and visual dysfunction [3].

Despite the generally stable appearance of the myelinated lesion, Straatsma syndrome predisposes affected eyes to severe vitreoretinal complications, most notably retinal detachment associated with macular hole formation [2, 4]. The pathophysiology of retinal detachment in this context differs from that observed in conventional high myopia. In Straatsma syndrome, firm vitreoretinal adhesion at sites of myelinated nerve fibers, particularly at the macula, generates tangential and anteroposterior tractional forces. When combined with chorioretinal atrophy and posterior staphyloma typical of high myopia, these forces favor macular hole formation and subsequent retinal detachment [2].

The present case describes a rhegmatogenous retinal detachment arising through a mechanism distinct from that classically reported in Straatsma syndrome, as it was caused by a peripheral retinal break in the absence of a macular hole. Although the coexisting high axial myopia represents a well‐recognized independent risk factor for peripheral retinal breaks through established mechanisms such as lattice degeneration and progressive peripheral retinal thinning, the intraoperative observation of abnormally strong vitreoretinal adhesion at the peripheral break site—in the absence of visible myelinated nerve fibers in that region—raises the hypothesis that vitreoretinal interface abnormalities in Straatsma syndrome may extend beyond areas of clinically apparent myelination. This single observational report is intended to generate a hypothesis rather than to establish a causal relationship.

## 2. Case Report

A 64‐year‐old man presented with a recent‐onset, rapidly progressive decline in visual acuity in the right eye. He had a longstanding diagnosis of Straatsma syndrome, characterized by persistent myelinated retinal nerve fibers involving the peripapillary region and extending along the major vascular arcades, with sparing of the mid‐ and far‐peripheral retina, amblyopia, and axial myopia in the right eye. The condition had been clinically followed for several years and, at prior examinations, best corrected visual acuity (BCVA) was 20/150 in the right eye, whereas the left eye consistently maintained a BCVA of 20/20. His ocular history was also notable for primary open‐angle glaucoma, adequately controlled with topical timolol maleate 0.5%. The systemic medical history was otherwise unremarkable, apart from well‐controlled systemic hypertension, and there was no contributory family history.

**Figure 1:**
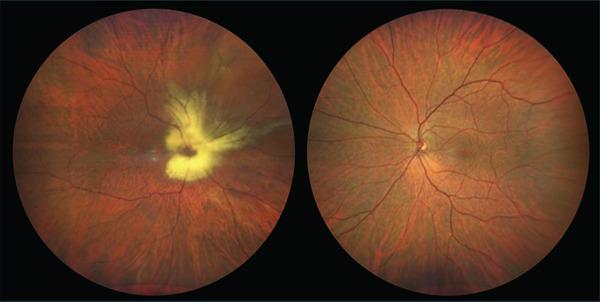
Preoperative color fundus photography showing extensive myelinated nerve fibers in the peripapillary region and along the proximal portions of the vascular arcades of the right eye. A superonasal retinal detachment is evident, with reduced macular clarity and mild vitreous haze. The left eye shows no abnormalities.

At presentation, BCVA in the right eye had decreased to hand motions, with stable 20/20 vision in the left eye. Slit‐lamp examination of the right eye disclosed a moderate nuclear cataract. Axial length measurements were 27.04 mm in the right eye and 24.68 mm in the left eye, consistent with axial anisometropic myopia. Funduscopic evaluation revealed a rhegmatogenous retinal detachment involving the superonasal quadrant of the right eye, extending to the macula (Figure 1).

**Figure 2:**
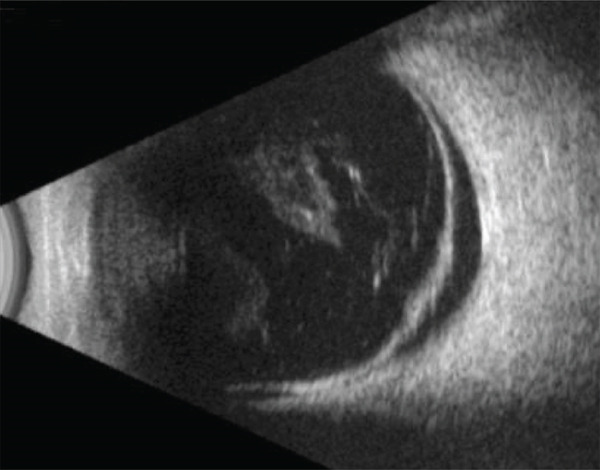
Preoperative superonasal longitudinal B‐scan ultrasonography of the right eye demonstrating retinal detachment extending to the macular region. Vitreous strands with vitreoretinal adhesions are observed in the midperipheral retina.

The B‐scan ultrasonography confirmed a superonasal retinal detachment with subretinal fluid extending toward the macula, with abnormally firm vitreoretinal adhesion notably in the midperiphery corresponding to the retinal detachment as well as adjacent vitreous condensations (Figure 2). Macular hole–related retinal detachment was considered but excluded based on clinical fundus examination.

Given the anatomic complexity and high‐risk profile associated with axial myopia and anomalous vitreoretinal adhesion, the patient underwent combined phacoemulsification with intraocular lens implantation and 25‐gauge pars plana vitrectomy, complemented by scleral buckling and 5000‐cSt silicone oil tamponade. Intraoperative findings demonstrated abnormally firm vitreoretinal adhesion at the site of the peripheral retinal break and, therefore, silicone oil tamponade was deliberately selected to enhance retinal stabilization and reduce the risk of postoperative complications.

**Figure 3:**
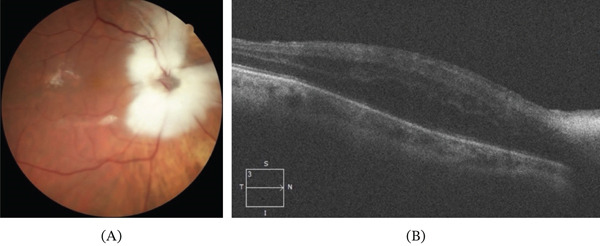
Imaging obtained 3 months after the primary surgical procedure, showing an attached retina at the posterior pole with (A) silicone oil tamponade and optical coherence tomography demonstrating (B) intraretinal macular edema. Increased optical reflectivity of the retinal nerve fiber layer is observed in the peripapillary region, corresponding to the area of myelination.

**Figure 4:**
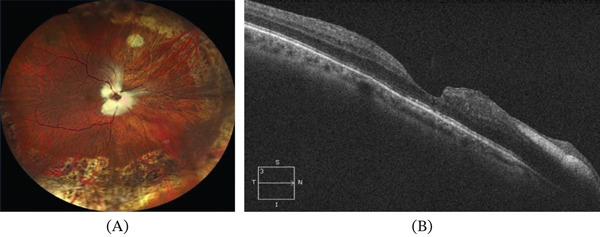
Final postoperative outcome 3 months after the second intervention (silicone oil removal and intravitreal dexamethasone implant), demonstrating (A) a fully attached retina with adequate peripheral indentation and peripheral laser scars, and (B) optical coherence tomography showing a macula without edema, with distortion of the foveal contour and persistence of myelinated nerve fibers in the peripapillary region.

In the early postoperative period, the retina remained fully attached. However, the patient developed chronic cystoid macular edema (Figure 3) after a 3‐month period, refractory to topical treatment with corticosteroid and nonsteroidal anti‐inflammatory drops, and a second procedure was performed, consisting of silicone oil removal and intravitreal implantation of a sustained‐release dexamethasone implant. Subsequent follow‐up demonstrated complete resolution of the macular edema, despite foveal contour distortion and stable retinal reattachment (Figure 4).

Three months after the second intervention, BCVA in the right eye improved to 20/60, a functional outcome considered consistent with the visual limitation imposed by pre‐existing amblyopia, which is known to constrain visual recovery despite successful anatomical repair. No adverse or unanticipated events were observed during follow‐up period.

## 3. Discussion

Straatsma syndrome has traditionally been regarded as a rare congenital condition with a largely static course, classically defined by the triad of myelinated retinal nerve fibers, ipsilateral axial myopia, and amblyopia [3, 5, 6]. Most reports emphasize its benign nature and focus predominantly on visual prognosis related to amblyopia rather than on progressive structural complications. Nevertheless, accumulating evidence suggests that the syndrome may involve subtle but clinically relevant abnormalities of the vitreoretinal interface, predisposing affected eyes to late‐onset vitreoretinal pathology, including retinal detachment [2, 4]. Although such associations remain uncommon, their recognition is crucial, as they expand the clinical spectrum of Straatsma syndrome beyond a purely developmental and functionally stable disorder.

The anomalous persistence of retinal nerve fiber myelination is thought to result from ectopic migration of oligodendrocytes beyond the lamina cribrosa during ocular development, a structure that normally acts as a barrier to intraocular myelination [1]. Beyond producing the characteristic ophthalmoscopic appearance, this aberrant myelination alters the microstructural and biomechanical properties of the inner retina. Several authors have demonstrated that areas of myelination are associated with thickening of the nerve fiber layer and abnormal vitreoretinal adhesion, likely related to alterations in glial architecture and extracellular matrix composition [2, 6]. These changes may have important mechanical implications, particularly during posterior vitreous detachment, by transmitting abnormal tractional forces to the underlying retina.

Previously reported cases of retinal detachment in Straatsma syndrome describe a mechanism centered on macular hole formation, typically in the context of high axial myopia [2, 4]. In these cases, strong vitreoretinal adhesion at the site of macular myelinated fibers has been implicated in the generation of tangential and anteroposterior traction, culminating in macular hole–associated retinal detachment. Surgical reports consistently emphasize the technical difficulty of inducing posterior vitreous detachment in these eyes, underscoring the strength of vitreoretinal adhesion overlying myelinated fibers [2, 4]. Consequently, the prevailing view in the literature is that retinal detachment in Straatsma syndrome is largely secondary to macular pathology rather than to peripheral retinal breaks.

In contrast to the macula‐centered mechanisms most commonly described in the literature, the present case documents a rhegmatogenous retinal detachment originating from a peripheral retinal tear in the absence of a macular hole, representing a less frequently recognized presentation. This observation raises the possibility that abnormal vitreoretinal adhesion in Straatsma syndrome may not be confined to the macular or peripapillary regions but may instead extend into the peripheral retina. However, because this inference is based on a single case, it remains speculative and requires corroboration from additional reports or histopathological studies.

It is essential to acknowledge that the patient′s demographic and biometric profile—a 64‐year‐old man with an axial length of 27.04 mm—already confers substantial independent risk for peripheral rhegmatogenous retinal detachment through well‐established mechanisms, including lattice degeneration, progressive peripheral retinal thinning, age‐related posterior vitreous detachment, and vitreous liquefaction [7]. High axial myopia alone is one of the strongest risk factors for peripheral retinal breaks, and this alternative explanation must be weighed before invoking a syndrome‐specific pathophysiological mechanism. In the present case, it is not possible to determine with certainty whether the peripheral break resulted primarily from myopia‐related degenerative changes, from a putative Straatsma syndrome–related vitreoretinal adhesion abnormality, or from a combination of both factors acting synergistically. The principal observation that raises the hypothesis of a syndrome‐specific contribution is the intraoperative finding of unusually firm vitreoretinal adhesion at the site of the peripheral retinal tear, in a region devoid of visible myelinated nerve fibers. However, several important caveats must be considered. First, the assessment of vitreoretinal adhesion firmness during vitrectomy is inherently subjective, operator‐dependent, and lacks any standardized or objective measurement method. Second, this observation cannot be independently verified, as no intraoperative recording, quantitative traction assessment, or histopathological analysis was performed. Third, vitreoretinal adhesion may also be abnormally firm in highly myopic eyes without Straatsma syndrome, particularly at sites of lattice degeneration or vitreous base pathology. Consequently, the proposed association between the intraoperative finding and Straatsma syndrome remains speculative rather than demonstrated. Nevertheless, the observation merits documentation for several reasons. In previously published cases of retinal detachment associated with Straatsma syndrome, multiple surgical teams have independently reported abnormally strong vitreoretinal adhesion overlying myelinated fibers, predominantly at the macula [2, 4]. The present case extends this pattern by noting a similar intraoperative impression at a peripheral site without clinically visible myelination, which—if corroborated in future reports—could suggest a more diffuse alteration of the vitreoretinal interface. It remains unknown whether subclinical myelination or myelination‐associated glial and extracellular matrix alterations exist at sites beyond ophthalmoscopically visible plaques, a question that can only be addressed by histopathological or advanced imaging studies.

Taken together, these considerations suggest that, in the setting of axial myopia‐related peripheral retinal fragility, abnormally strong vitreoretinal adhesion—whether related to myelination‐associated changes or occurring through as yet undefined mechanisms—may contribute to the development of peripheral retinal tears and subsequent rhegmatogenous retinal detachment, independent of macular hole formation. Although this proposed mechanism cannot be established from a single case, the observation broadens the range of pathogenic pathways that should be considered in patients with Straatsma syndrome and warrants further investigation.

From a clinical perspective, recognition of this alternative presentation carries important implications. It emphasizes the need for meticulous examination of the peripheral retina and long‐term surveillance in patients with Straatsma syndrome, particularly in the presence of high axial myopia, even when the macula appears structurally intact. Furthermore, surgeons should anticipate the possibility of unusually strong vitreoretinal adhesion extending beyond clinically visible myelinated regions, a factor that may influence surgical planning and intraoperative decision‐making.

This study has several limitations that must be acknowledged. First, as a single case report, these findings are inherently hypothesis‐generating and cannot establish a causal relationship between Straatsma syndrome and the observed peripheral retinal detachment. The intraoperative assessment of vitreoretinal adhesion firmness is subjective and was not corroborated by objective measurement, intraoperative video documentation, or histopathological analysis. Additionally, the peripheral retinal break could not be documented on preoperative color fundus photography, and preoperative optical coherence tomography of the peripheral break site was not available, precluding detailed structural characterization of the vitreoretinal interface at that location. Finally, the patient′s coexisting high axial myopia represents a major confounding factor, as it independently predisposes to peripheral retinal breaks through well‐established mechanisms, and it is not possible to definitively attribute the observed findings to Straatsma syndrome rather than to myopia alone.

In conclusion, the present case documents a peripheral, tear‐related rhegmatogenous retinal detachment in a patient with Straatsma syndrome, a presentation distinct from the macular hole–driven process most commonly reported in this condition. Although the coexisting high axial myopia precludes definitive attribution of the peripheral break to syndrome‐specific vitreoretinal abnormalities, the intraoperative observation of unusually firm vitreoretinal adhesion at a site devoid of visible myelinated fibers raises the hypothesis that vitreoretinal interface alterations in Straatsma syndrome may be more diffuse than previously appreciated. This observation is hypothesis‐generating and requires corroboration from additional case reports, surgical series, and, ideally, histopathological studies. Nevertheless, it reinforces the importance of meticulous peripheral retinal examination and long‐term surveillance in patients with Straatsma syndrome, particularly in the presence of high axial myopia.

## Funding

No funding was received for this manuscript.

## Ethics Statement

The study adhered to the principles outlined in the Declaration of Helsinki. The protocol was approved by the Institutional Review Board (IRB No. 92887125.1.8707). Written informed consent was obtained from the patient for publication of this case report and any accompanying images.

## Conflicts of Interest

The authors declare no conflicts of interest.

## Data Availability

All data generated or analyzed during this study are included in this article. Further inquiries can be directed to the corresponding author.
